# Spatial heterogeneity of type I error for local cluster detection tests

**DOI:** 10.1186/1476-072X-13-15

**Published:** 2014-05-27

**Authors:** Aline Guttmann, Xinran Li, Jean Gaudart, Yan Gérard, Jacques Demongeot, Jean-Yves Boire, Lemlih Ouchchane

**Affiliations:** 1 Department of Biostatistics, Medical Informatics and Communication Technologies, Clermont University Hospital, Clermont-Ferrand F-63000, France; 2 UMR CNRS UDA 6284 ISIT, Auvergne University, Clermont-Ferrand F-63001, France; 3 UMR 912 SESSTIM (INSERM IRD AMU), Aix-Marseille University, Marseille F-13005, France; 4 Assistance Publique Hôpitaux de Marseille, Biostatistic and Modelisation, Marseille F-13005, France; 5 La Tronche University School of Medicine, FRE CNRS 3405 AGIM, J. Fourier University, Saint-Martin-d’Hères F-38700, France

**Keywords:** Cluster detection test, Type I error, Simulation study, Edge effect, Spatial scan

## Abstract

**Background:**

Just as power, type I error of cluster detection tests (CDTs) should be spatially assessed. Indeed, CDTs’ type I error and power have both a spatial component as CDTs both detect and locate clusters. In the case of type I error, the spatial distribution of wrongly detected clusters (WDCs) can be particularly affected by edge effect. This simulation study aims to describe the spatial distribution of WDCs and to confirm and quantify the presence of edge effect.

**Methods:**

A simulation of 40 000 datasets has been performed under the null hypothesis of risk homogeneity. The simulation design used realistic parameters from survey data on birth defects, and in particular, two baseline risks. The simulated datasets were analyzed using the Kulldorff’s spatial scan as a commonly used test whose behavior is otherwise well known. To describe the spatial distribution of type I error, we defined the participation rate for each spatial unit of the region. We used this indicator in a new statistical test proposed to confirm, as well as quantify, the edge effect.

**Results:**

The predefined type I error of 5% was respected for both baseline risks. Results showed strong edge effect in participation rates, with a descending gradient from center to edge, and WDCs more often centrally situated.

**Conclusions:**

In routine analysis of real data, clusters on the edge of the region should be carefully considered as they rarely occur when there is no cluster. Further work is needed to combine results from power studies with this work in order to optimize CDTs performance.

## Abstract (fr)

**Résumé:**

**Contexte**

Les tests de détection de clusters (CDT) permettent à la fois de détecter et de localiser les clusters. Au même titre que pour la puissance, il est donc nécessaire d’étudier la répartition spatiale de l’erreur de type I de ces CDT. Dans le cas de l’erreur de type I, la répartition spatiale des clusters détectés à tort (WDC) peut être particulièrement concernée par un effet de bord. Cette étude de simulation a pour objectif de décrire la distribution spatiale des WDCs et de confirmer et quantifier la présence de cet effet de bord.

**Méthodes**

Ce travail s’appuie sur la synthèse de 40 000 jeux de données simulant l’hypothèse nulle d’homogénéité spatiale des risques. Les simulations étaient fondées sur les paramètres réels de données d’un registre de malformations congénitales, et notamment sur deux risques de base réels. La description de la distribution spatiale de l’erreur de type I nous a conduits à définir le concept de taux de participation de chaque unité spatiale de la région. Cet indicateur a ensuite été intégré pour la construction d’un nouveau test statistique destiné à confirmer et quantifier l’effet de bord.

**Résultats**

La valeur globale de l’erreur de type I à 5% a bien été retrouvée. Les résultats montraient un très net effet de bord avec un gradient décroissant du taux de participation depuis le centre vers le bord, les WDC étant plus souvent situés en zone centrale.

Lors de la mise en œuvre des CDT sur données réelles, les détections de clusters près du bord d’une région d’étude doivent être examinées avec la plus grande attention, ces dernières étant très rares en l’absence de cluster réel. Il est maintenant nécessaire d’orienter de futurs développements vers la combinaison de ces résultats à ceux des études de puissance, et ce dans le but d’optimiser les performances des CDT.

## Background

Spatial clusters can be detected using a wide range of statistical tests [1,2] many of which are available in free software such as R [3,4]. Epidemiologists use cluster detection tests (CDTs) to detect clusters without *a priori* knowledge either of their number or their location, and to determine their significance. CDTs performance being a function of epidemiological and geographical context [1,5-11], it is recommended to perform power studies before using these tests in a particular region for a given phenomenon. However, statistical power is not the only test characteristic determining performance. Performance at large depends on two type of risks: type I and type II errors.

In presence of clusters, usual statistical power (1-β) is not sufficient to assess CDT performance to reject the null hypothesis of risk homogeneity. At worst, a CDT could have a maximum power to reject this null hypothesis of risk homogeneity but never correctly locate the true cluster. Similar concern can be raised for type I error. A CDT could, under the null hypothesis of no cluster, generate wrongly detected clusters (WDC) preferentially localized in particular zones of the studied region. The overall type I error could effectively be equal to its predefined value usually set to 5%, but the interpretation of the analyses would certainly not be the same for detected clusters inside or outside such zones.

In the case of statistical power, authors have since used either evaluation of power and location by different indicators [6,12-14] or concomitant evaluation of both with a single measure such as the extended power [15,16]. The development of single measure of performance taking into account both power and location accuracy has enabled systematic spatial evaluation of performance on entire regions [15]. The question of the spatial evaluation of CDT is, so far, not totally answered with regards to power because evaluation of factors such as relative risks or cluster shape and size are still assessed by a non-systematic approach based on more or less arbitrary settings in simulation designs.

The question of relative risks and clustering characteristics is not relevant in the spatial evaluation of type I error, other factors have to be taken into account, however. First, there is still one epidemiological factor that requires setting: the baseline risk. For an applicative purpose, the use of the baseline incidence of the studied disease is the evident choice, but for research, a systematic evaluation over a wide range of this factor should be carried out. Second, simulation studies evaluating type I error are much more likely to be influenced by edge effect [17-19] than power studies. Indeed, in the majority of simulation studies assessing power, edge effect is largely lessened by designs simulating clusters wholly within the studied region.

We aimed to evaluate CDTs regarding the spatial distribution of type I error. Such description was carried-out at the level of the spatial unit (SU) introducing the concept of SU’s participation rate. We proposed a statistic to quantify and test for edge effect which was of particular interest. We used Kulldorff spatial scan statistic as an example of CDT, whose behavior is otherwise well known, and performed a simulation study using realistic parameters from survey data on birth defects.

## Methods

### Disease modeling

The study region was the Auvergne region (France), divided into *n* = 221 spatial units (SUs) equivalent to U.S. ZIP codes. We applied two baseline risks (incidences) of birth defects to the same at-risk population, whose size was approximated by mean annual number of live births.

For a realistic analysis, we used data archived in CEMC (birth defects registry for the Auvergne region) and INSEE (National Institute of Statistics and Economic Studies) databases. We collected two categories of data from 1999 to 2006: all birth defects and cardiovascular birth defects. Both datasets were sorted by SU. The number of live births was approximated by the number of birth declarations in the at-risk population. Global annual incidences of all birth defects (I_all_) and cardiovascular birth defects (I_cv_) were estimated at 2.26% and 0.48% of births, respectively.

### Datasets

We generated 20 000 datasets for each baseline risk, *i.e.* a total of 40 000 datasets.

Each dataset is entered as a table of 221 rows and 5 columns. The rows contain the coordinates (longitude and latitude) of a SU, the observed number of cases, the size of the at-risk population (*i.e.*, the number of live births) and the expected number of cases in the specified SU. This last quantity is the product of the global incidence (I_all_ or I_cv_) and the at-risk population size in the SU. The observed case numbers are assumed as independent Poisson variables such that

$$E\left(N_{i}\right)=\mu_{i},N_{i}\sim \mathrm{Pois}\left(\mu_{i}\right),i=1,\ldots ,n$$

where *N*_*i*_ is the observed number of cases, and *μ*_*i*_ denotes the expected number of cases in the *i*th SU under the null hypothesis of risk homogeneity.

### Assessment of type I error

#### *Overall rate*

The global type I error rate was estimated by the proportion of WDC over the 20 000 datasets for each baseline risk.

#### *Spatial distribution*

*SU participation rate:* Participation rate of each WDC in the overall type I error is equal to *1/m*, with *m* the number of WDCs. Participation rate of each SU in the overall type I error was estimated by a weighted sum of the number of times each SU was included in a WDC. This weight is a function of *m* and the length of each WDC (number of SUs within). For each SU *i* among the *n* SUs of the region, the participation rate *P*_*i*_ in the overall type I error is such that

$$P_{i}=\overset{m}{\underset{j=1}{\sum }}I_{\mathrm{ij}}{\left({\mathrm{ml}}_{j}\right)}^{-1}$$

where *m* is the number of WDCs, *l*_*j*_ is the length of the *j*th WDC and I_*ij*_ a binary indicator equal to 1 when the *i*th SU is within the *j*th WDC and 0 otherwise. By construction, *P*_*i*_ ≥ 0 and $\;\overset{n}{\underset{i=1}{\sum }}P_{i}=1$, where *n* is the number of SUs in the region.

**Figure 1:**
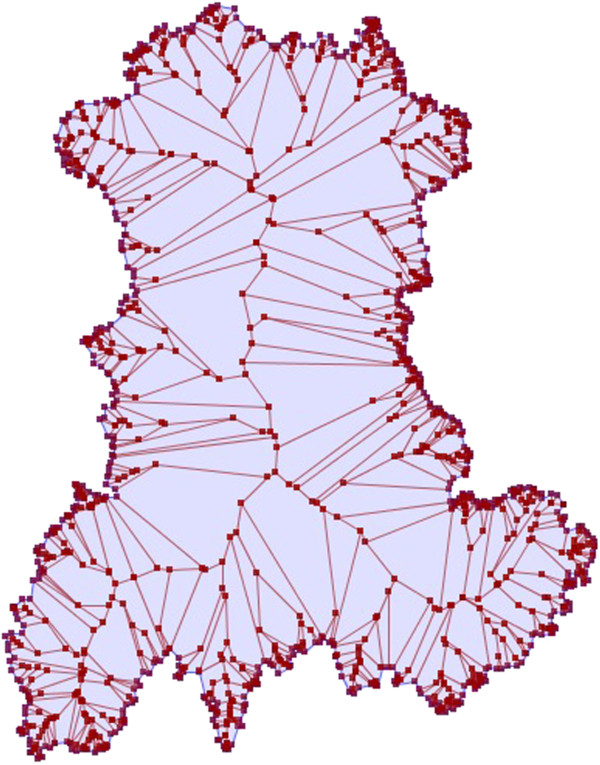
Representation of the medial axis of the Auvergne region.

*Edge effect:* The edge effect is defined here as an inhomogeneous distribution of *P*_*i*_ characterized by a gradient from the medial axis (or cut locus or skeleton) of the region to its edge. This gradient can either be ascending or descending. The medial axis is the set of all points having more than one closest point on the region’s edge [20-23]. The Figure 1 shows the medial axis of the region under study^a^. For such a simple polygon, the medial axis is a tree whose leaves are the vertices and whose edges are straight segments reflecting local symmetries of the shape.

To confirm the presence of an edge effect, we propose a test whose statistic, referred to as *E*, is such that

$$\left\{\begin{array}{c} Ε=\overset{n}{\underset{i=1}{\sum }}\epsilon_{i}\left(P_{i}-n^{-1}\right)\\ \epsilon_{i}=\left(1-\frac{2d_{i}}{D}\right) \end{array}\right)$$

Where *d*_*i*_ is the minimal Euclidian distance between the centroid of the *i*th SU and the edge of the region, *D* the maximum Euclidian distance between any point of the medial axis and the region closest edge, and *n* the number of SUs in the region. By construction, as 0 ≥ *d*_*i*_ ≥ *D*, -1 ≥ ϵ_*i*_ ≥ +1. The coefficient ϵ_*i*_ is a continuous indicator quantifying how much a point can be considered “on the edge” of the region. It is referred to as “the edge coefficient” in the remainder of this paper. For any point in the region, the closer to the edge, the higher the edge coefficient, and the closer to the medial axis, the smaller the edge coefficient. The edge coefficient ranges from -1 for the most “central/medial” points of the region to +1 for points on the edge. For a study region divided into census tract, each SU is attributed the edge coefficient of its centroid. All SUs with the same edge coefficient are at the same distance to the edge and the closer to the medial axis, the smaller the edge coefficient, tending to -1 for the most “central” SUs of the region.

The test hypotheses are expressed by

$$\left\{\begin{array}{c} H_{0}:E=0\\ H_{1}:E\neq 0 \end{array}\right)$$

The quantity *n*^*-1*^ is the expected participation rate for all SUs under the null hypothesis of spatial homogeneity in type I error. When *P*_*i*_ is higher than expected towards the edge of the region, by construction, it is lower towards the center (as $\overset{n}{\underset{i=1}{\sum }}P_{i}=1$) and there is an ascending gradient. On the contrary, when *P*_*i*_ is higher towards the center of the region, there is a descending gradient. The statistic *E* is positive when there is an ascending gradient of *P*_*i*_ and negative when the gradient is descending. Indeed, in case of an ascending gradientand *E* will tend to be highly positive.

• central SUs will tend to have

(1) $$\epsilon_{i}<0,\left(P_{i}-n^{-1}\right)<0$$

• border SUs will tend to have

(2) $$\epsilon_{i}>0,\left(P_{i}-n^{-1}\right)>0$$

In case of a descending gradientand *E* will tend to be highly negative.

• central SUs will tend to have

(3) $$\epsilon_{i}<0,\left(P_{i}-n^{-1}\right)>0$$

• border SUs will tend to have

(4) $$\epsilon_{i}>0,\left(P_{i}-n^{-1}\right)<0$$

Finally, under H_0_ of spatial homogeneity of type I error, the sum of all *P*_*i*_, equal to 1, is homogeneously distributed among the n SUs with an expected participation rate equal to n^-1^. Under this null hypothesis, the expected value of (*P*_*i*_ - *n*^- 1^) is null and independent to *ϵ*_*i*_. Consequently, under null hypothesis, the expected value of *E* is null.

Since the variance of the *E* statistic under H_0_ (spatial homogeneity of type I error) is unknown, we used Monte Carlo simulation where the n observed *P*_*i*_ were randomly distributed 99 999 times among the n SUs in the region. The p-value was the proportion of elements among the collection of simulated and observed statistics which were greater than or equal to the observed value. The precision of this p-value was thus of 10^-5^ digits.

### Kulldorff’s spatial scan statistic

In this study, we selected Kulldorff’s spatial scan statistic [24,25] as a well-known and widely used CDT whose performance has been studied by many authors [1,6,10,26]. The spatial scan statistic detects the most likely cluster on locally observed statistics of likelihood ratio tests. The scan statistic considers all possible zones *z* defined by two parameters: a center that is successively placed on the centroid of each SU, and a radius varying between 0 and a predefined maximum. The true geography being delineated by administrative tracts, each zone *z* defined by all SUs whose centroids lie within the circle, is irregularly shaped. Let *N*_*z*_ and *n*_*z*_ be respectively the size of the at-risk population and the number of cases counted in zone *z* (over the whole region, these quantities are the total population size *N* and the total number of cases *n*). The probabilities that an at-risk case lies inside and outside zone z are respectively defined by *p*_*z*_ *= n*_*z*_*/N*_*z*_ and *q*_*z*_ *= (n-n*_*z*_*)/(N-N*_*z*_*)*. Given the null hypothesis of risk homogeneity H_0_: *p*_*z*_ *= q*_*z*_, versus the alternative H_1_: *p*_*z*_ *> q*_*z*_ and assuming a Poisson distribution of cases, Kulldorff defined the likelihood ratio statistics as proportional to

$${\left(\frac{n_{z}}{{\mathrm{\lambda N}}_{z}}\right)}^{n_{z}}{\left(\frac{n-n_{z}}{\lambda \left(N-N_{z}\right)}\right)}^{n-n_{z}}Ι\left[n_{z}>{\mathrm{\lambda N}}_{z}\right],$$

where *λ* (here equal to I_all_ or I_cv_ depending on the case considered) is the global incidence and the indicator function *I* equals 1 when the number of observed cases in zone *z* exceeds the expected number under H_0_ of risk homogeneity, and 0 otherwise. The circle yielding the highest likelihood ratio is identified as the most likely cluster. The p-value is obtained by Monte Carlo inference.

Over the 40 000 simulated datasets, each test was performed with a maximum size of zone z set to 50% of the total at-risk population, a number of 999 Monte Carlo samples for significance measures, and an alpha level set to 5%.

### Software

Data simulation and analysis were performed on R 2.14.0 [3,27-29], using the function “kulldorff” of the SpatialEpi package [27] to perform the Kulldorff’s spatial scan.

## Results

### Overall rate and WDC characteristics

**Figure 2:**
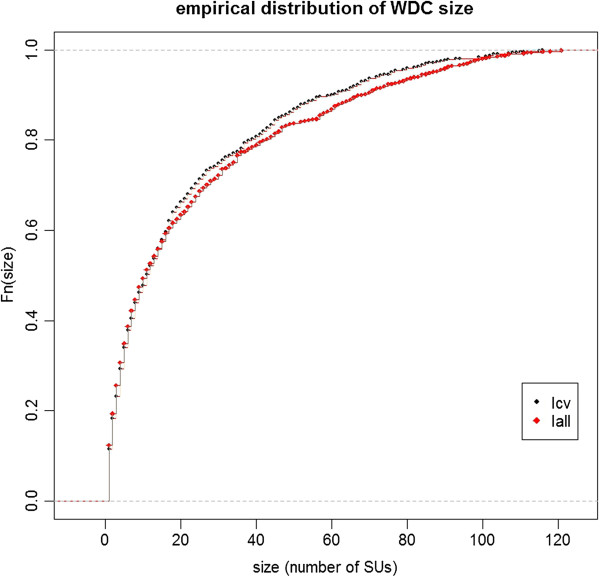
Empirical distribution of WDC size (number of SUs) over 20 000 simulated datasets for two baseline incidences of birth defects: 2.26% (Iall) and 0.48% (Icv).

The overall type I error rate was 5.11% (1021 WDC over 20 000 datasets; CI 95% [4.80%, 5.42%]) for I_all_ and 5.06% (1012 WDC over 20 000 datasets; CI 95% [4.76%, 5.38%]) for I_cv_. The average size of WDCs was 21.4 SUs (minimum 1SU, median 11 SUs, maximum 116 SUs) and 23.4 SUs (minimum 1SU, median 11 SUs, maximum 132 SUs), respectively. The Figure 2 shows the empirical distribution of the WDC size for each baseline risk.

### SUs participation rates

**Figure 3:**
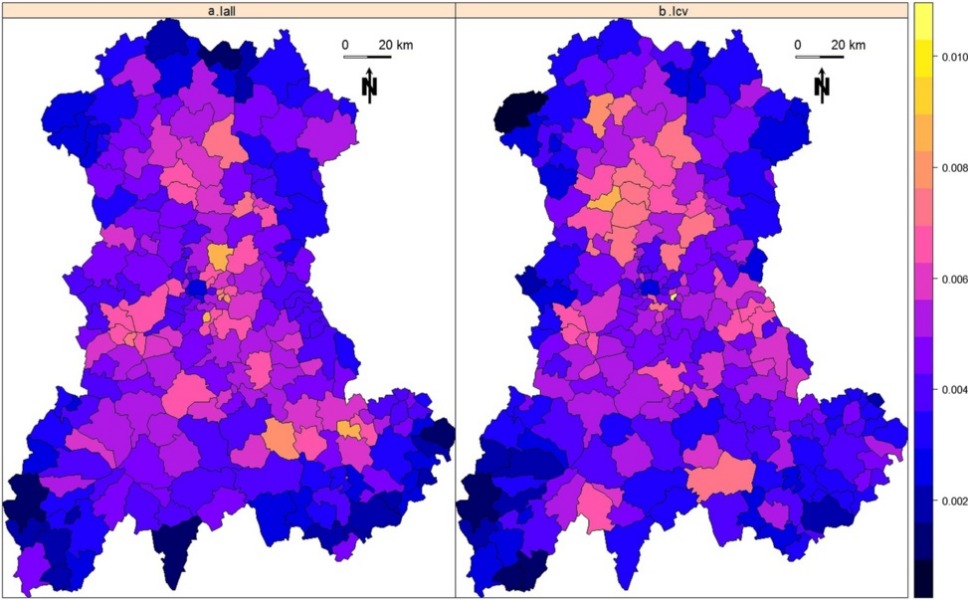
**SUs participation rates computed over 20 000 simulated datasets for each map. (a)** Observed values for baseline incidence of birth defects set to 2.26% (Iall). **(b)** Observed values for baseline incidence of birth defects set to 0.48% (Icv).

**Figure 4:**
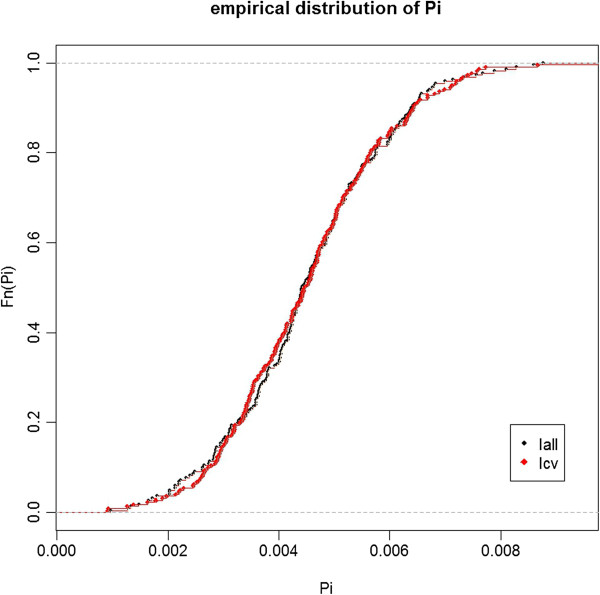
Empirical distribution of SUs participation rates computed over 20 000 simulated datasets for two baseline incidences of birth defects: 2.26% (Iall) and 0.48% (Icv).

Figure 3 shows the SUs participation rates for baseline risks I_all_ (Figure 3a) and I_cv_ (Figure 3b). The expected participation rate (*n*^*-1*^) for each SU is equal to 0.452%. With 0.452% ± 0.147% (mean ± standard deviation) for I_all_ and 0.452% ± 0.148% for I_cv_, the two observed distributions of participation rates were very close to each other (Figure 4). The observed values varied from 0.097% to 0.877% for I_all_ and from 0.091% to 1.03% for I_cv_.

**Figure 5:**
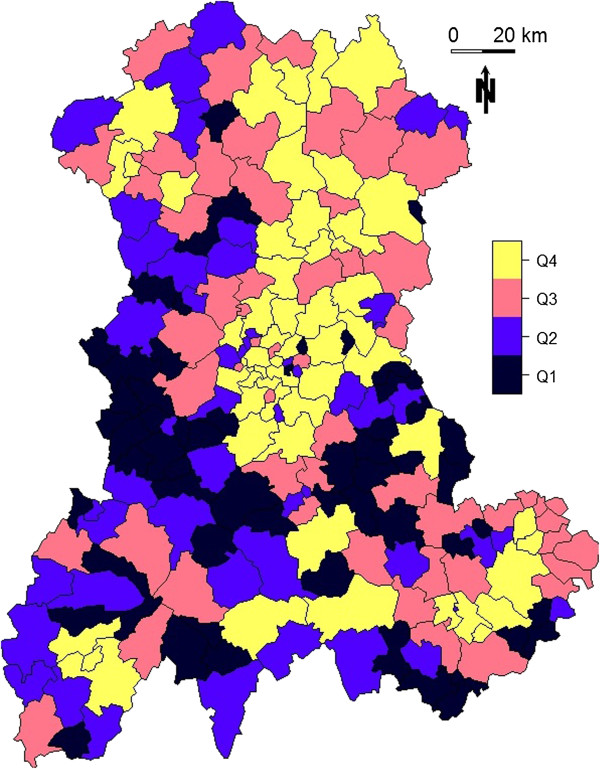
**Size of the at-risk population for each SU in the Auvergne region, as defined by mean number of live births per year between 1999 and 2006 (source: INSEE).** Q1: ≤ 17; Q2: > 17 and ≤ 35; Q3: > 35 and ≤ 70; Q4: > 70.

We sought for a correlation between *P*_*i*_ and size of the at-risk population (Figure 5) by Spearman’s rank test. Both coefficients were negative but none resulted in significant relationship (r = -0.13 with p-value = 0.056 for I_all_ and r = -0.11 with p-value = 0.1 for I_cv_).

### Edge effect

**Figure 6:**
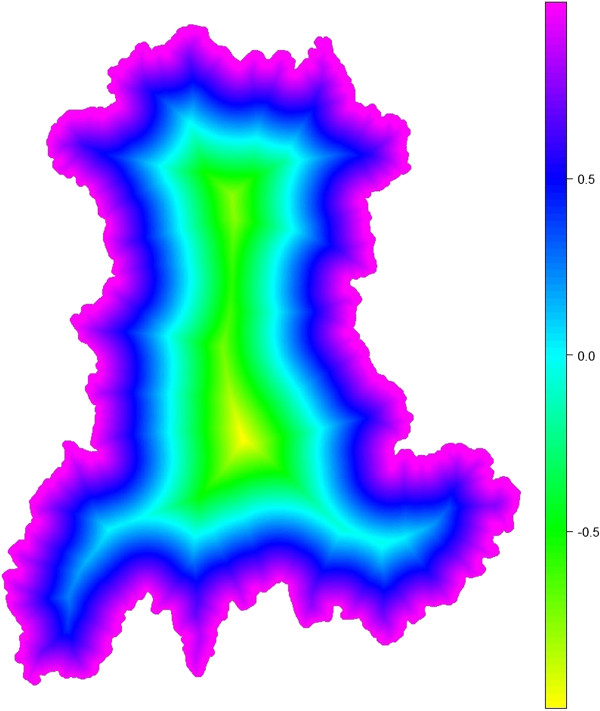
Values of the edge coefficient ϵi computed over a regular sampling of 500 000 points within the region.

**Figure 7:**
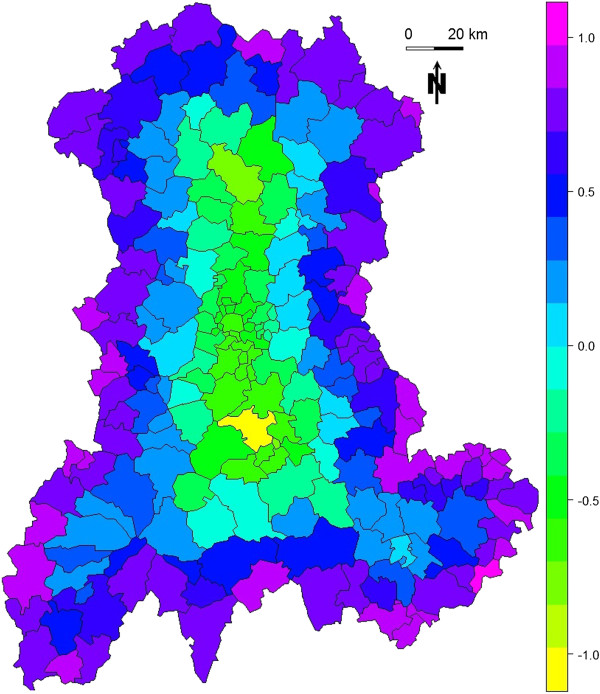
**Values of the edge coefficient ϵi computed for each SU within the region.** Each SU is assigned the value of the edge coefficient ϵi computed for its centroid.

Figure 6 shows the value of the edge coefficient ϵ_*i*_ computed for a regular sampling of 500 000 points within the region. Figure 7 shows the value of the edge coefficient computed for the *n* = 221 SUs within the region.

With *E* equal to -0.086 for I_all_ and -0.074 for I_cv_, both simulations resulted in descending gradient of *P*_*i*_, *i.e.* higher *P*_*i*_ for central SUs. As shown by *E* values, this gradient was stronger for I_all_ than for I_cv_.

**Figure 8:**
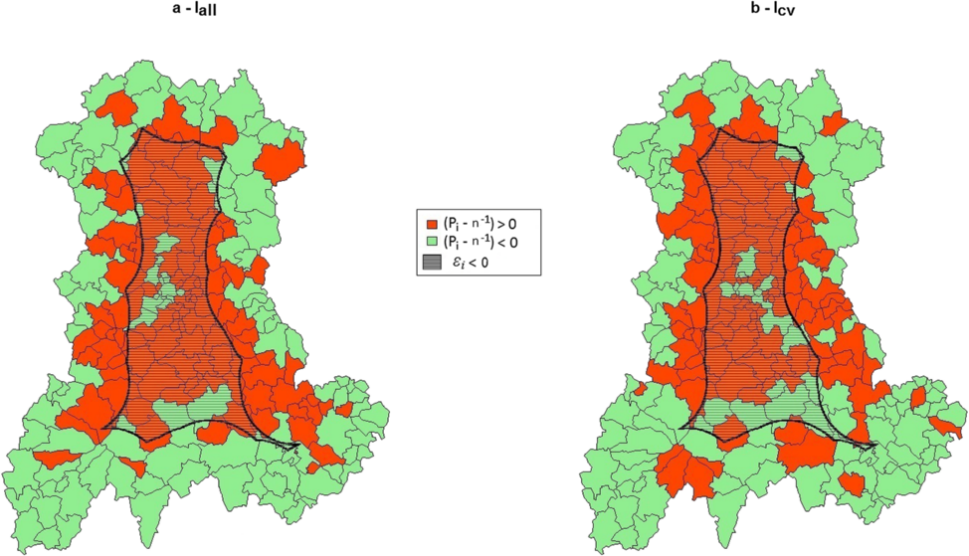
**Overlaying of the sign of the difference between observed and expected values of SUs participation rates (computed over 20 000 simulated datasets for each map) and the sign of the edge coefficient ϵi (ϵi negative in hatched area). (a)** Baseline incidence of birth defects set to 2.26%. **(b)** Baseline incidence of birth defects set to 0.48%.

As shown in Figure 8, the SUs contributing to the overall type I error for more than *n*^*-1*^ (*P*_*i*_ > *n*^*-1*^) were mostly located away from the border of the region. The black line delineates a central zone where the edge coefficient is negative and a complementary zone where the edge coefficient is positive. Within the central zone, red SUs contribute negatively to *E* (see Equation 3), on the contrary, outside the central zone, red SUs contribute positively to *E* (see Equation 4).

**Figure 9:**
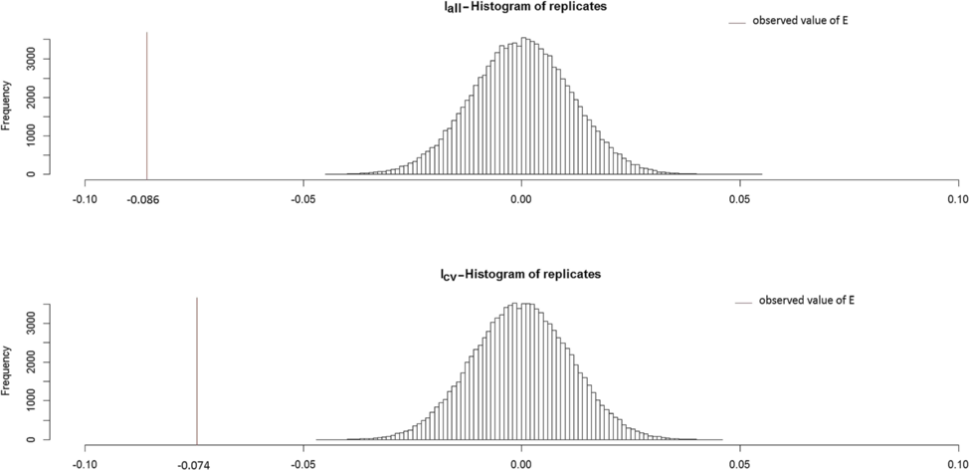
Histograms of the null distribution (99 999 replicates) and observed values of E for two baseline incidences of birth defects: 2.26% (Iall) and 0.48% (Icv).

Both tests were highly significant, with Monte Carlo p-values both equal to 10^-5^ (99 999 replicates). Figure 9 shows the simulated null distributions of *E* and the observed values for the two simulated baseline risks.

## Discussion

We have shown that type I error is heterogeneously distributed with a descending gradient from center to edge. Even if global type I error is very near the predefined 5%, WDCs are rarely located on the edge of the map. In a survey system, where sensitivity matters over specificity, it could be argued that since global type I error is preserved, the global cost in unfruitful secondary investigation is not affected by the spatialization of type I error.

 Our work did not aim to test for clustering in type I error rate and thus we did not used CDTs to analyze the spatial distribution of *P*_*i*_. We note, however, that methods such as Bayesian smoothing could be of interest in the description of the spatial distribution of type I error. As the presence of an edge effect with descending gradient was obviously expected, our contribution aimed to describe, quantify and test for this edge effect. Furthermore, within a given region, the spatial description of type I error makes possible to see with precision which detected clusters should be carefully considered because they are less likely to coincide with false alarm.

The edge effect was present and strong, no matter the baseline risk. Only two levels have been tested for this risk. One could wonder about a possible correlation between edge effect and the level of baseline risk. Levels at regular interval between these two baseline risks are currently being explored and there is no evidence of such a correlation so far (data not shown).

The edge effect is indisputable in this study (Figure 8) and the statistic *E* has consequently resulted in a highly significant test. This statistic is based on the edge coefficient *ϵ*_*i*_ that defines what is “on the edge” of the map and what is not. By using medial axis, we proposed a distance-based definition, but other parameters could be considered. For instance, it could be useful to distinguish between two SUs at the same distance to the edge but in different configurations with one in a “peninsula” (between two edges) and thus more isolated than the others. To be accounted for, this factor needs geometrical tools to characterize the spatial isolation.

Aside from a purely geometrical definition of what is an edge, confounding factors should also be taken into account. Suppose that the at-risk population is heterogeneously distributed, with more populated areas centrally localized. Then, suppose again that the at-risk population size is negatively correlated to participation rate (this was not the case in our study). Our test for edge effect might turn out to be significant, concluding in an ascending gradient of *P*_*i*_ from center to edge, only due to this confounding factor. In our simulations, the at-risk population is effectively more centrally localized. If the negative correlation between population size and *P*_*i*_ had been significant, we would have an even stronger evidence for a descending edge effect regarding *P*_*i*_ from center to edge, because our results, that turned out to be significant, would have actually been underestimated.

Even if we did not find any relationship between population size and participation rate, other factors (such as the number of neighbors, the accessibility by road or rail system, *etc.*) should be evaluated. The best way to deal with these confounding factors might be to integrate them in the construction of ϵ_*i*_ for geographical factors or to replace the constant n^-1^ by a vector of expected participation rates for epidemiological factors. For the *E* statistic to be equal to 0 under H_0_ (spatial homogeneity of type I error), this last adaptation should be done in such a way that the sum of all expected participation rates stays equal to 1.

Our results highlight the edge effect in type I error, and thus can help the interpretation of real data analysis. It could be even more useful to provide a way to integrate spatial heterogeneity of type I error in the analysis itself. Furthermore, adjustment in CDT behavior should be done to address this issue only if it does not impede the tests’ power. In a previous simulation study on CDT performance, we proposed a method to build performance map based on a systematic spatial evaluation [15]. The now available data for both H_1_ (single clusters of 4SUs in this previous study) and H_0_ (risk homogeneity) in similar settings (same baseline risk and population size) will enable us to study whether and how it could be gainful to add a spatial adjustment of type I error.

## Conclusion

Spatial heterogeneity of type I error should be considered when interpreting analysis of real data, because of the strong edge effect. This work clearly shows that a detected cluster on the edge of the region of interest is less common when no alarm should be raised. To explore all avenues, assessment of edge effect and its factors, as well as development of tools to integrate it in routine health survey, should be considered.

## Endnotes

^a^Computation of the straight skeleton was performed using [30] and the results were imported and displayed with JTS Topology Suite [31], a software under GNU license.

## Abbreviations

WDC: Wrongly detected cluster; CDT: Cluster detection test; H_0_: Null hypothesis; H_1_: Alternative hypothesis; I_all_: Incidence of all birth defects; I_cv_: Incidence of cardiovascular birth defects.

## Competing interests

The authors declare that they have no competing interests.

## Authors’ contributions

AG and LO conceived the design, performed the study and drafted the manuscript. AG was responsible for statistical programming and data analysis. YG contributed to the construction of the *E* test. JD, JG, YG, XL and JYB contributed to manuscript revision. All authors read and approved the final manuscript.
